# Fibrin structure in organized thrombotic material removed during pulmonary artery endarterectormy: the effect of vessel calibre

**DOI:** 10.1007/s11239-016-1382-z

**Published:** 2016-06-02

**Authors:** Piotr Mazur, Bogusław Gawęda, Joanna Natorska, Michał Ząbczyk, Anetta Undas, Jerzy Sadowski, Grzegorz Kopeć, Marcin Waligóra, Piotr Podolec, Bogusław Kapelak

**Affiliations:** Institute of Cardiology, Jagiellonian University Medical College, Kraków, Poland; The John Paul II Hospital, Kraków, Poland

**Keywords:** Chronic thromboembolic pulmonary hypertension, Thrombus architecture, Pulmonary arteries, Fibrin

## Abstract

Pulmonary endarterectomy (PEA) is a curative therapeutic approach in patients with chronic thromboembolic pulmonary hypertension (CTEPH). The location-dependent structural differences of thrombotic material found in pulmonary arteries in CTEPH are poorly investigated. We present the case of a 47-year-old woman with antiphospholipid syndrome, diabetes mellitus and abnormal fibrin phenotype, who underwent PEA for CTEPH. Intravascular material removed bilaterally during PEA (from lobar, segmental and sub-segmental arteries) has been studied using light and scanning electron microscopy (SEM). Light microscopy showed tighter fibrous network in the portions of intraluminal thrombotic material facing the vessel wall, which contained collagen and fibrin fibers, and abundant cells. Cells, evaluated by immunostaining, were present in the whole removed material. Tissue factor expression was also observed with the highest values in the portions of intravascular material facing the vessel wall. In the main pulmonary arteries, SEM images revealed thick fibers of fibrous proteins loosly meshed and few erythrocytes and platelets between them (both dysmorphic “wedged” and fresh cells were present). In the fibrotic layers, containing mainly collagen and fibrin, removed from the lobar/segmental pulmonary arteries we found a stepwise increase in fiber density with decreasing vessel calibre, followed by denser fibrous networks composed of thinner fibers. Elastic fibers in the lobar and segmental arteries were aligned along the blood flow vector. These findings demonstrate differences in the structure of endarterectomized PEA material dependent on the vessel calibre and might contribute to understanding of CTEPH pathophysiology.

## Introduction

Chronic thromboembolic pulmonary hypertension (CTEPH) follows almost 4 % of pulmonary embolism (PE) cases [1]. Pulmonary endarterectomy (PEA) is a treatment of choice [2]. In case of endogenous thrombolytic potential failure, thromboembolus can become organized in lungs through fibroblast invasion, becoming adherent to the vessel wall [3]. Microscopically, matrix deposition, mixed cellularity and variable small vessel recanalization is noticed [4].

Patients with CTEPH are characterized by impaired fibrinolysis due to alterations in fibrin(ogen) structure, affecting accessibility to plasmin cleavage sites [5]. Fibrin clots in CTEPH are lysis-resistant (N-terminal fragments of the fibrin beta-chain are released) [6]. Also in the setting of antiphospholipid syndrome (APS), unfavourable fibrin phenotype and denser clots are observed [7]. Prothrombotic states and impaired clot dissolution are believed to contribute to CTEPH development in APS [8].

Previously, we reported increasing fibrin content and fibrin network density with the pulmonary arteries diameter decrement in acute PE thrombus [9].

Herein we present a case of a 47-year-old woman with CTEPH, APS and abnormal fibrin properties, who underwent PEA in our institution. The obtained material was studied with multimodal assessment of cellular and acellular contents in light microscopy and scanning electrone microscopy (SEM).

## Case report

A 47-year-old woman (body mass index, 35.2 kg/m^2^) with CTEPH, a non-smoker with type 2 diabetes mellitus (DM) on oral medication (gliclazide + metformin), gastrointestinal reflux disease, parafrenia and a history of deep venous thrombosis (DVT) in the left lower extremity with subsequent recurrent PE was admitted to our institution in December 2014 due to worsening of dyspnea and exertion intolerance. Last documented episode of DVT followed by PE occured in July 2013; the treatment with low molecular weight heparin was then initiated and continued until June 2014, when oral anticoagulation with warfarin was started. Recommended INR values were 2.0–3.0, but the time within therapeutic range was 63 %. Due to gradual clinical detoriation, she underwent evaluation with pulmonary angiography and computed tomography. While hospitalized, the patient presented with exertional dyspnea (4.4 METs on ergospirometry with modified Naughton protocol). Transthoracic echocardiogram demonstrated right ventricular (RV) overload and distention with moderate tricuspid valve insufficiency, RV systolic pressure exceeding 85 mmHg and elevated pulmonary pressure with RV outflow tract acceleration time of 78 ms. Left ventricular systolic function was preserved (left ventricle ejection fraction was 70 %) and RV systolic function was mildly impaired (tricuspid annulus systolic excursion was 20 mm). The right heart catheterization revealed mean PA pressure of 40 mmHg and mean pulmonary artery wedge pressure of 5 mmHg.

**Fig. 1:**
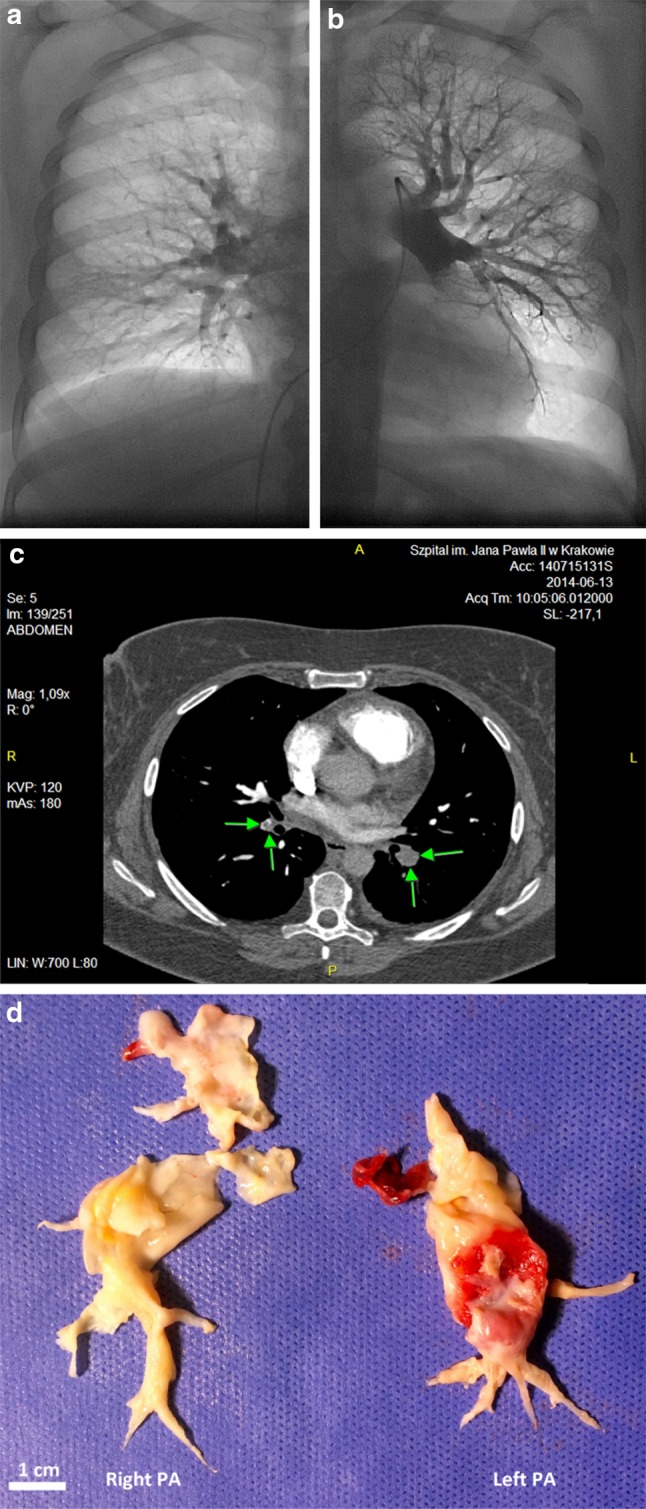
Pulmonary angiogram, computed tomography and intraoperative images. Pulmonary right (**a**) and left (**b**) angiogram showing deficient perfusion, more pronounced in the right pulmonary artery (**a**). Contrast-enhanced computed tomography showing thrombotic material in lower-lobe branches of right and left pulmonary arteries (**c**). Intraoperative picture of the thrombotic material removed from pulmonary arteries during PEA (**d**), *scale bar* 1 cm

Pulmonary angiogram showed deficient perfusion in both lungs, with amputated left lower-lobe pulmonary artery and many “webs” in branches of both pulmonary arteries (Fig. 1a, b). Contrast-enhanced computed tomography (CT) revealed embolic material in the lower-lobe branches of right and left pulmonary arteries and in their segmental branches (Fig. 1c). Smaller amounts of embolic material were also visualized in the segmental branches of both upper lobar pulmonary arteries, and at the branching point of the right middle lobar artery. Lower extremities ultrasound excluded current DVT, and the patient underwent PEA, which was performed in December 2014.

The PEA was performed with extracorporeal circulation and temporary circulatory arrest in deep hypothermia. Both pulmonary arteries were opened. Organized thrombotic material (Jamieson Type II [10]) was surgically removed from lobar and segmental branches of the right and left pulmonary artery (from segments A2, A3, A4–A5, A6–A10 and A4, A5, A6–A10, respectively) (Fig. 1d). The patient required prolonged ventilatory support and intensive respiratory rehabilitation during the post-operative period. During an uneventful 11-month-long follow-up, she received a warfarin treatment. The clinical work-up revealed acquired thrombophilia (family history was negative towards thrombosis) and APS, which was diagnosed based on the modified APS classification criteria by Miyakis et al. [11]. Estimation of lupus anticoagulant was performed using a clot-based assay according to the 2009 recommendations [12]. Anticardiolipin and anti-β2GP-I antibodies were determined by immunoenzymatic assays (INOVA Diagnostics, San Diego, USA). Reference ranges for IgG were up to 15 GPL and 8 SGU, respectively, and for IgM up to 17 MPL and 10 SMU, respectively. The diagnosis was reevaluated after 12–16 weeks [13]. The following auto-immunology workup was non-specific and did not confirm any other systemic auto-immune disease. We excluded cancer as a cause of thrombosis. Fibrin phenotype analysis after 11-months-long follow-up (on warfarin) was done as previously described [14]. Despite normal fibrinogen level (2.74 g/l), we found strongly decreased fibrin clot permeability (Ks = 4.05 ± 0.16 10^−9^cm^2^ using a previously described methodology [15]), as compared with healthy controls from our previous report (n = 30, Ks = 7.55 [7.00–7.96]) 10^−9^cm^2^ [14]).

**Fig. 2:**
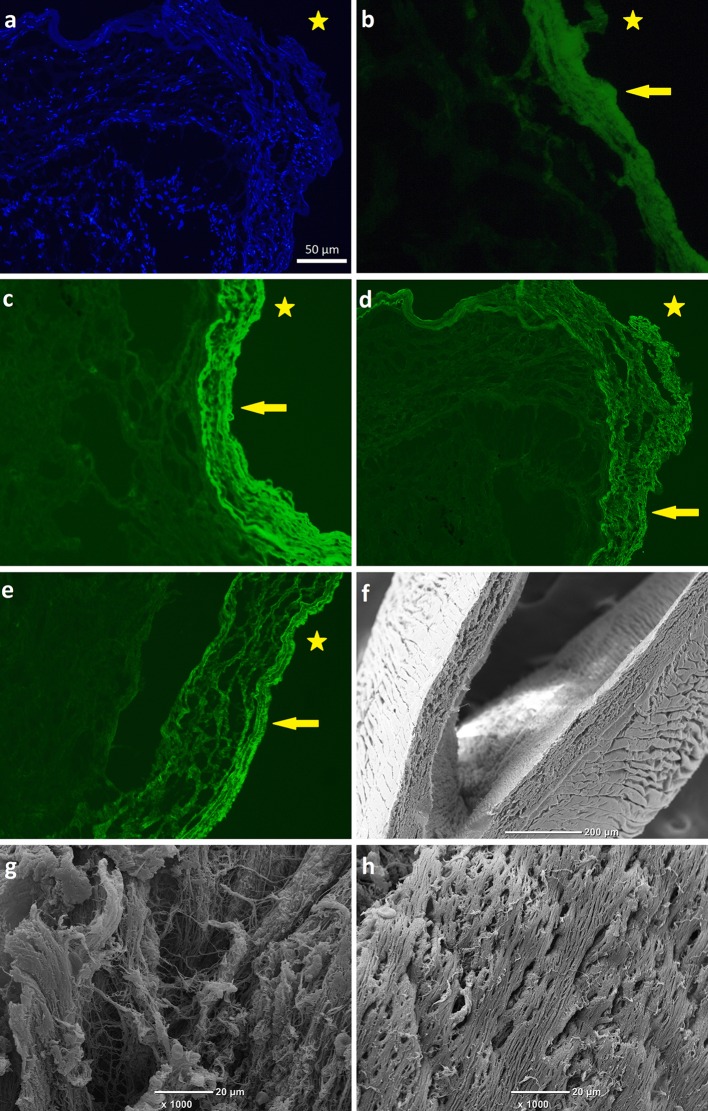
Representative immunostaining and scanning electron microscopic (SEM) images. Representative micorphotgraphs of thrombi immunostaining showing **a** cellular structure of thrombi. Cells nuclei stained with DAPI (*blue*); **b** fibrin immunostaining (*green*), collagen immunostaining of **c** small vessels and **d** large vessels (*green*); **e** tissue factor immunostaining (*green*). *Arrows* indicate positive staining; *stars* indicate abluminal section; **a**–**e** magnification ×40, *scale bar* 50 μm. Representative SEM images of endarterectomized material from the right distal pulmonary artery; **f** the right proximal pulmonary artery; **g** and distal right segmental artery; **h** showing different fiber compaction; **f** magnification ×100, *scale bar* 200 μm. **g**–**h** Magnification ×1000; *scale bar* 20 μm

The resected material from both arteries was divided into sections to multimodally investigate its morphology across the specimens (from most proximal through most distal parts). All sections were investigated with all modalities. Firstly, the analysis utilizing hematoxylin-eosin staining performed. It revealed abundant cells influx and the presence of fibrous tissue in all sections. Immunostaining showed the accumulation of collagen and fibrin and identified the cells as macrophages (anti-CD68+staining) and fibroblasts, dispersed all over the sections (Fig. 2a). Coexisting fibrin (Fig. 2b) and collagen (Fig. 2c, d) were found explicitly on the surface of the intravascular material facing the vessel wall, forming an uniform layer in all specimens from small-calibre and large-calibre pulmonary vessels. Furthermore, we performed a collagen fibers density measurement, in which the distance between two adjacent fibres was assessed and expressed as a mean of 15 single measurements ± standard deviation. We observed an enhanced collagen fibers density (1.426 ± 0.76 μm) in small-calibre vessels, as compared with the large-calibre vessels (3.378 ± 1.94 μm) [Fig. 2c, d, respectively, the depicted differences reflect samples from the small-calibre (distal) vs. large-calibre (proximal) vessels chosen macroscopically during the dissection of the endarterectomized material]. Tissue factor (TF) staining revealed its presence within both luminal (facing the vessel lumen) and abluminal (facing the vessel wall) areas, however TF accumulation was prominent within abluminal parts of the thrombus, corresponding to tighter fibrills network structure (Fig. 2e).

The thrombotic material was also evaluated using SEM, as previously described [16]. After washing, the thrombus was fixed with 2.5 % glutaraldehyde phosphate buffered saline solution. Specimens were dehydrated, gold coated, and photographed digitally with a JEOL JSM 5410 (JEOL, Tokyo, Japan). The SEM images revealed some fresh thrombotic material in the left PA and fibrous layers as the effect of chronic thrombosis both in the left and right PA (Fig. 2f–h). Fibers in the lobar and segmental (right and left) arteries were aligned along the blood flow vector. The thrombus structure from the right PA was characterized by thick fibers and large pores between them. Few erythrocytes and platelets (both dysmorphic “wedged” and fresh cells) were observed between the fibrous layers from large arteries. In the fibrotic layers removed from the lobar/segmental (right and left) pulmonary arteries we found a stepwise increase in fibers density followed by thinner fibers and smaller pores between them (Fig. 2h). The elastic fibers layers that coated distal parts of the segmental (right and left) arteries were much more compressed than in the proximal parts.

## Discussion

This study demonstrates that the structure of intravascular material in CTEPH is more compact in distal pulmonary arteries than in proximal ones. We report that within the intraluminal material in CTEPH, the compaction of fibrin polymer network and TF expression are increased in the sections facing the vessel wall.

Wall shear rate and wall shear stress are increased in smaller arteries, and the final result of thrombosis depends on interplay between rheodynamics and clot properties under flow, determined largely by fibrin [17]. Thrombus degradation efficiency depends on fibrin network characteritics as well [18]. Fibrin clots from CTEPH patients are lysis resistant, as demonstrated in 2006 by Morris et al. [6]. Recently, Vikerfors et al. demonstrated lower Ks values and denser fibrin clots in APS patients [7]. Type 2 DM [19, 20] is also associated with lysis-resistant fibrin. Both APS and DM are frequently present in CTEPH patiens [21, 22]. In our patient, impaired fibrin clot characteristics cannot be explained by an increase in fibrinogen level, which is an important determinant of Ks, but rather by alterations in fibrin(ogen) structure. Abnormal fibrin phenotype, together with physical factors (different flow characteristics in vessels of smaller diameter, as compared with larger arteries), might determine the creation of dense and linear network of fibers in vessels of smaller calibre. Arguably, this can determine the structure of thrombotic material already early in the course of the disease, and enable thrombolysis failure despite the anticoagulation [9].

Fibrin not only forms the structural scaffold for thromboembolus, but can also activate PA endothelial cells [23] contributing to vascular remodeling. Fibrin-mediated increase of interleukin-8 expression facilitates migration of repair cells along the endothelial cells by interaction with intracellular adhesion molecule type 1 [24] and macrophage-1 antigen [25] receptors, which might partially elucidate fibrin-macrophages collocalization presented by us.

In our intravascular specimens, TF expression was observed predominantly in the abluminal sections (facing the vessel wall), and fibrous mesh was denser in those parts. In CTEPH, the endothelial cells adjacent to in situ thrombi tightly attached to vessel walls were found to express high level of plasminogen activator inhibitor type 1 [26]. The presence of more compact fibrous network in the abluminal portions might either elicit or reflect the vasculopathic processes involved in CTEPH. Further investigation is needed to confirm whether the structure of intravascular material in CTEPH is uniform among the patients. The CTEPH development itself, at least in some cases, is likely connected to abnormal fibrin phenotype, which under favourable physical conditions has the potential to produce a lysis-resistant clot.

In conclusion, this report shows that intravascular material in CTEPH is more dense in small-calibre distal pulmonary arteries. Fibrin network density and TF expression are increased close to the vessel wall in vessels of any calibre. The fibers in the intraluminal material in CTEPH are aligned along the flow vector in pulmonary arteries. Further investigation is needed to confirm these observation in a larger group of CTEPH patients.
